# Computational View toward the Inhibition of SARS-CoV-2 Spike Glycoprotein and the 3CL Protease

**DOI:** 10.3390/computation8020053

**Published:** 2020-05-31

**Authors:** Zhen Qiao, Hongtao Zhang, Hai-Feng Ji, Qian Chen

**Affiliations:** 1 Department of Orthopaedics, The Warren Alpert Medical School of Brown University, Rhode Island Hospital, Providence, RI 02903, USA;; 2 Departments of Pathology and Laboratory Medicine, Perelman School of Medicine, University of Pennsylvania, Philadelphia, PA 19301, USA;; 3 Department of Chemistry, Drexel University, Philadelphia, PA 19104, USA

**Keywords:** COVID-19, coronavirus, protease, spike protein, computational, inhibition

## Abstract

Since the outbreak of the 2019 novel coronavirus disease (COVID-19), the medical research community is vigorously seeking a treatment to control the infection and save the lives of severely infected patients. The main potential candidates for the control of viruses are virally targeted agents. In this short letter, we report our calculations on the inhibitors for the SARS-CoV-2 3CL protease and the spike protein for the potential treatment of COVID-19. The results show that the most potent inhibitors of the SARS-CoV-2 3CL protease include saquinavir, tadalafil, rivaroxaban, sildenafil, dasatinib, etc. Ergotamine, amphotericin b, and vancomycin are most promising to block the interaction of the SARS-CoV-2 S-protein with human ACE-2.

## 1. Introduction

As of 24 May 2020, over 5 millions people in the world have been confirmed as having the 2019 novel coronavirus disease (COVID-19), an infection with Severe Acute Respiratory Syndrome coronavirus 2 (SARS-CoV-2) (initially called 2019-nCoV before 11 February 2020) which is part of the Coronaviridae family of positive-sense single-stranded RNA viruses that includes SARS-CoV and MERS-CoV (Middle East Respiratory Syndrome coronavirus), both of which also cause severe respiratory infections. The death count in China so far has been over 1700, but the number is expected to go higher with the increasing number of confirmed and non-confirmed cases. The medical research community is vigorously seeking a treatment to control the infection and save the lives of severely infected patients.

Just a few weeks after the COVID-19 outbreak, the complete genome of SARS-CoV-2 was determined and reported to GenBank (accession MN908947). Viruses were also isolated from patients to understand the genomic characteristics and mechanism of the viral infection. As revealed by the analysis, the SARS-CoV-2 shared 79% sequence identity to SARS-CoV. In one study, SARS-CoV-2 was found to be closely related to two bat-derived Severe Acute Respiratory Syndrome (SARS)-like coronaviruses, with 87.5% and 87.6% shared identity [1]. In another study, SARS-CoV-2 was 96% identical at the whole-genome level to a bat coronavirus [2].

Despite the high sequence identity between the SARS-CoV-2 and the SARS-CoV in the open reading frame regions, the envelop spike protein (S-protein) [3], which mediates the infection of SARS-CoV via the human host protein ACE-2, has only about 80% shared sequence identity between the SARS-CoV and SARS-CoV-2 [1]. Within the S-protein, the receptor docking domain has a higher divergence, with four out of five critical ACE-2 interacting amino acid residues replaced in the SARS CoV-2. However, structural modeling indicated that the four residues in the SARS-CoV-2 retain a structural conformation similar to that of SARS-CoV, and the SARS-CoV-2 S-protein should be able to bind ACE-2 with reasonable affinity^4^. Indeed, studies by Zhou et al. using cells expressing human ACE-2 confirmed that the SARS-CoV-2 could infect cells via the same protein on ACE-2 as SARS-CoV did [2]. Thus, one option to treat the infection is to search for an inhibitor that can prevent the interaction of the SARS-CoV-2 S-protein with human ACE-2. The availability of the genome sequence of SARS-CoV-2 allows us to establish structural models for the S-protein [4].

The RNA of coronaviruses encodes polyproteins that can be processed by viral proteases to yield mature proteins. The same mechanism is shared by picornaviruses and retroviruses. Patients treated with protease inhibitors appeared to have much better clinical outcomes than without using the inhibitors (SARS death: 28.8% vs. 2.4%) [5]. Molecular dynamics simulations have revealed that, by molecular docking to the active site of the main protease 3CL of SARS-CoV, both lopinavir and ritonavir could induce conformation changes and potentially interfere with infection by SARS virus [6]. We expect the same will apply for SARS-CoV-2. The crystal structure of the SARS-CoV-2 protease (3CL^pro^) was just recently reported by Liu et al. [7]. Thus, another option to treat the SARS-CoV-2 infection is to search for inhibitors of the SARS-CoV-2 3CL^pro^.

With these models and crystal data, we performed in silico studies of potential inhibitors of the SARS-CoV-2 S-protein and 3CL^pro^.

## 2. Computational Methods

All calculations were operated on Dell PowerEdge C6220 servers. The chemical structures were prepared by AutoDockTools-1.5.6 [8], Chimera 1.14 [9], and Avogadro [10]. The docking studies were performed with Autodock 4.2.6, Autodock4, AutoDockTools4 [11], and Autodock Vina 1.1.2 [12].

### 2.1. Preparation of Receptor and Ligands

The 3CL protease’s three-dimensional crystal structure was retrieved from the Protein Data Bank (PDB ID: 6LU7), and it was applied as the receptor for molecular docking after a cleaning with Chimera. The ligands observed, i.e., FDA-approved drugs (2454 structures in total), were retrieved from the BindingDB (https://www.bindingdb.org), and the structures of the ligands were further optimized with Avogadro. The force field applied for geometry optimization was MMFF94.

The SARS-CoV-2/ACE-2 structure was retrieved using the function of the comparative modeling of the Chimera interface with the modeler (version 9.23) [13]. For the preparation of the SARS-CoV-2/ACE-2 structure, the target template sequence was retrieved from Zhang et al.’s work and the SARS-CoV/ACE-2 (PDB ID: 6ACD) served as a template, as it was also the top candidate from Basic Local Alignment Search Tool (BLAST) results. Because SARS-CoV and SARS-CoV-2 have an 88% similarity, the 3D structure can be predicted with a high accuracy. Next, the sequence alignments were performed using SARS-CoV as a template. Then, the model was built followed by refining the loops, side chain optimization, and model optimization. When the homology model was generated, it was further validated using the WHATCHECK/PROCHECK program [14] for basic parameters like torsion angle, rotational angle, bond length, etc. Finally, this model was used as receptor for docking purposes. The loop refinement and side chain optimization were performed using Chimera 1.14 by selecting the active region; all the parameters were the default of the version.

**Figure 1.**
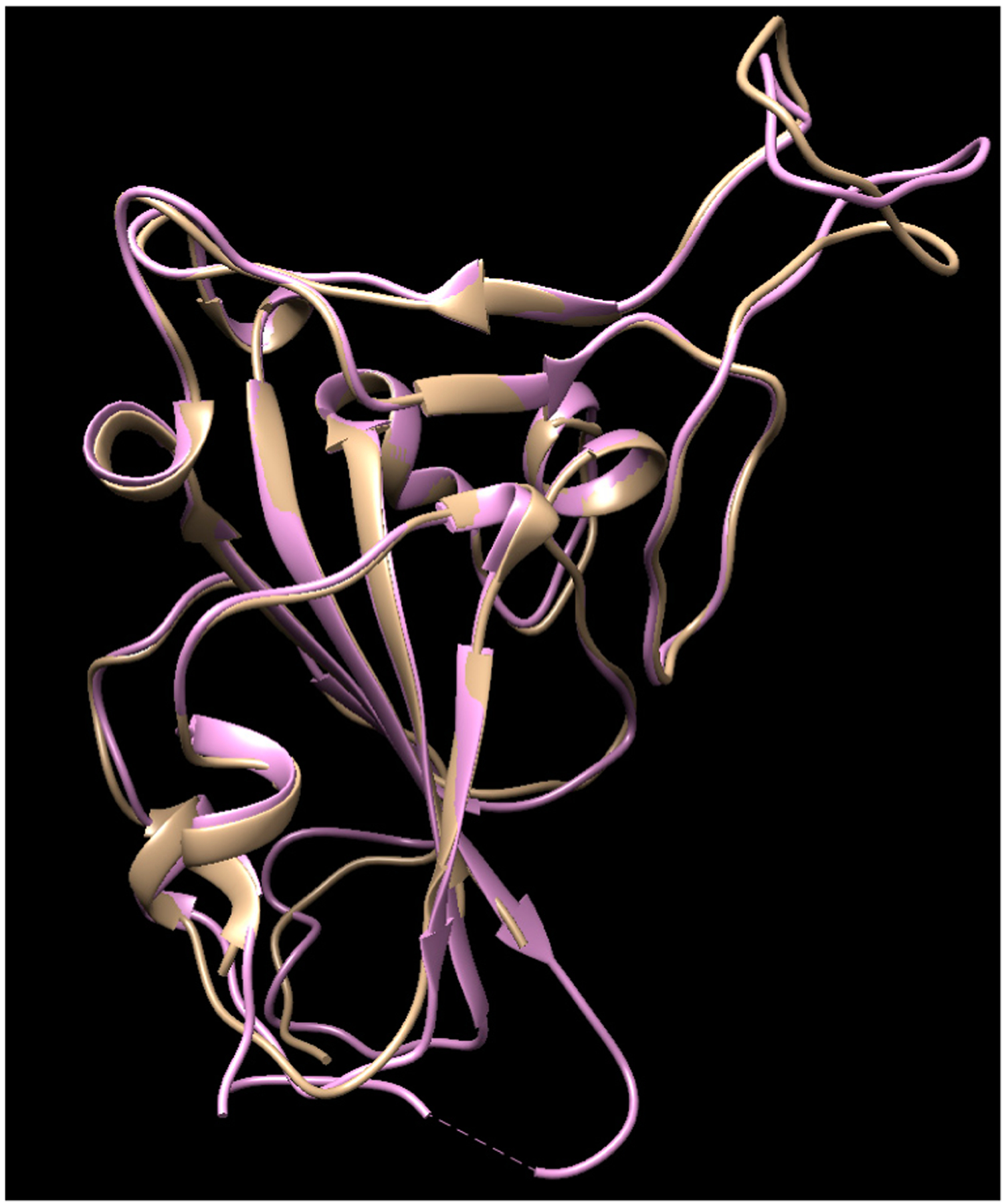
The comparison of the crystal structures of the SARS-2 spike protein (6VW1, pink color) with our homology modeling (light brown color) using the SARS-2 template.

It is noteworthy that this calculated work was performed before the crystal structure of the COVID-19 S-protein was released (6LZG, 6VW1, etc.). After the crystal structures were released, their structures were compared with ours and the structures overlaid well (Figure 1), with 93.22% of its residues in the allowed region and a minor difference on the top right loop, which was not a site that interacts with the ACE-2, so a re-calculation was not conducted using the new crystal structures.

### 2.2. Molecular Docking with Autodock Vina

For the SARS-CoV-2 3^CL^ inhibition calculation, the input files for Autodock Vina were prepared in the receptor’s original file (PDB format) and ligands files (SDF format) using AutoDockTools-1.5.6. After minimizing, the grid box was set at 22.00 Å × 22.00 Å ×22.00 Å along the x, y, and z axis, respectively. The docking site was defined at 1.00 Å when using the Autodock Vina. The grid box was set into the docking site at the H41, C145, and E166 regions according to the docking site of the coronavirus main proteinase (3^CL^) of Severe Acute Respiratory Syndrome (SARS). Then, the receptor file (PDBQT format, for docking purposes) was prepared by the addition of polar only hydrogen atoms, the removal of all water molecules, and the calculation of the Gasteiger charge. The instructed command prompts were used for the docking process. The docking output file includes the docking energy (in kcal/mol, which is an indication of the binding affinity/efficiency of one specific ligand to the receptor molecule) and the interaction of the ligands with the receptor (hydrogen bond, pi-pi stacking, etc.).

For the SARS-CoV-2 S-protein inhibition calculation, the PDB files of the SARS-CoV-2 S-protein were generated using the homology modeling method in Chimera; the template used for this was the SARS-CoV S-protein. After minimization, the input file was prepared using AutoDockTools-1.5.6. The grid box, which was a rectangular shaped area that covered all the possible docking sites of the SARS-CoV-2 S-protein with its receptor ACE-2, was chosen as 22.00 Å × 42.00 Å ×22.00 Å along the x, y, and z axis, respectively. The docking site was defined at 1.00 Å when using the Autodock Vina. Then, the receptor file (PDBQT format, for docking purposes) was prepared by the addition of polar only hydrogen atoms, the removal of all water molecules, and the calculation of the Gasteiger charge.

### 2.3. Analyzing the Docking Results with Chimera and BioLuminate

The docking results were ranked in the order from high to low in different modes according to the docking scores (docking energy, kcal/mol). The ligands with the most negative docking scores—i.e., the highest affinities—were selected for the visualization of the docked complexes using Chimera [9].

The docking energies of the SARS-CoV-2 S-protein and human ACE-2 were calculated using BioLuminate [15–17], and then compared to the docking energies of the SARS-CoV S-protein and human ACE-2. To verify whether those ligands can be used for blocking the interaction of the S-protein with human ACE-2, the docking energies of the SARS-CoV-2 S-protein/ligands and human ACE-2 were also calculated. The solvation model used was VSGB [18], and the force field chosen was OPLS_2005 [19] for all the docking energy predictions.

## 3. Results

### 3.1. Results of the SARS-CoV-2 3^CL^ Protease

**Figure 2.**
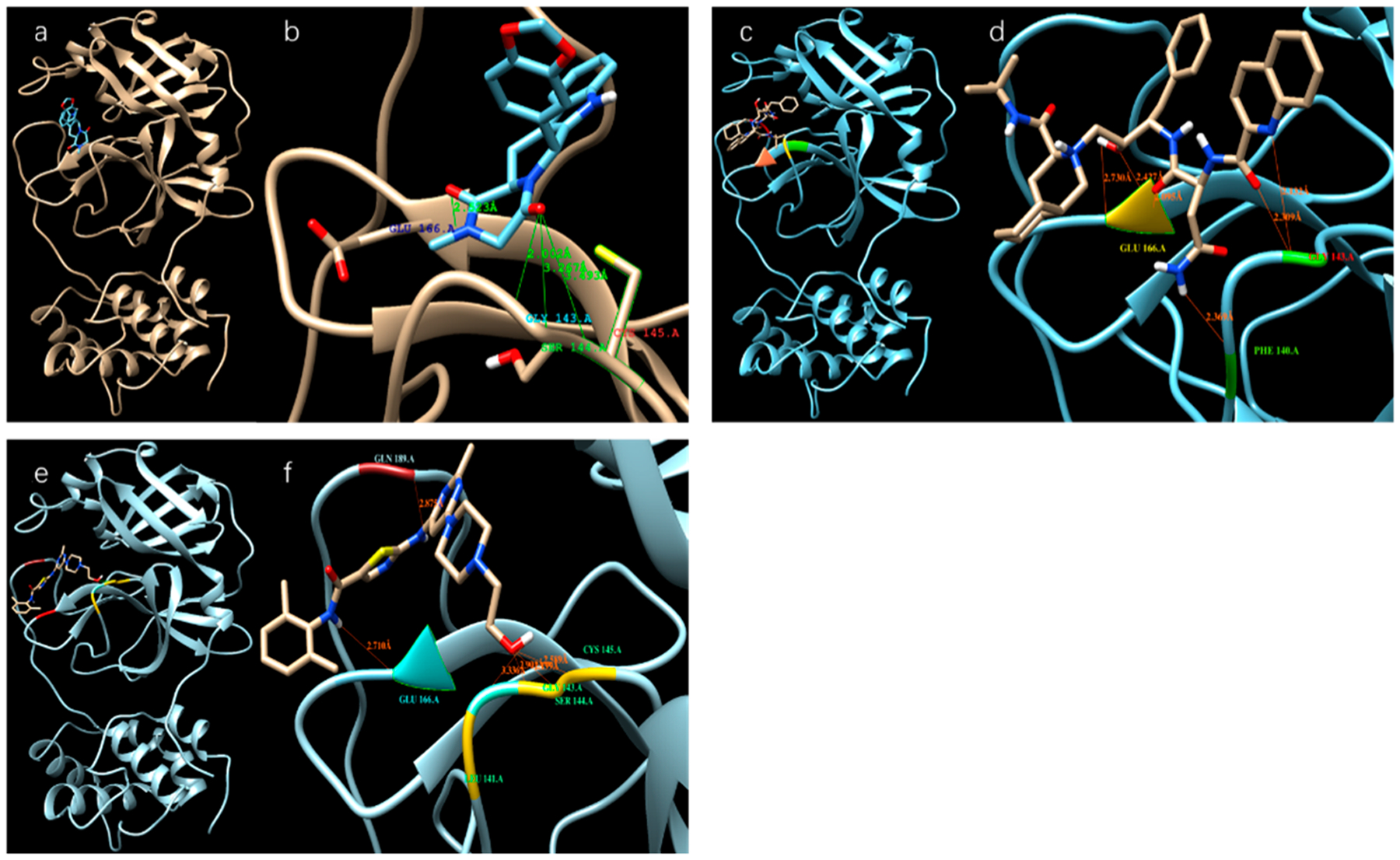
Different ligands in the docking site of SARS-CoV-2 protease. (**a**), (**b**) Tadalafil; (**c**), (**d**) Saquinavir; (**e**), (**f**) Dasatinib.

Table 1 shows the binding affinity of several ligands with SARS-CoV-2 3^CL^ protease sorted according to the docking scores (binding affinities) calculated from the Autodock Vina; Figure 2 shows the docking of those with high docking scores—Tadalafil, Dasatinib, and Saquinavir—with the protease in the docking sites of the protease.

### 3.2. Results of SARS-CoV-2 S-Protein

**Figure 3.**
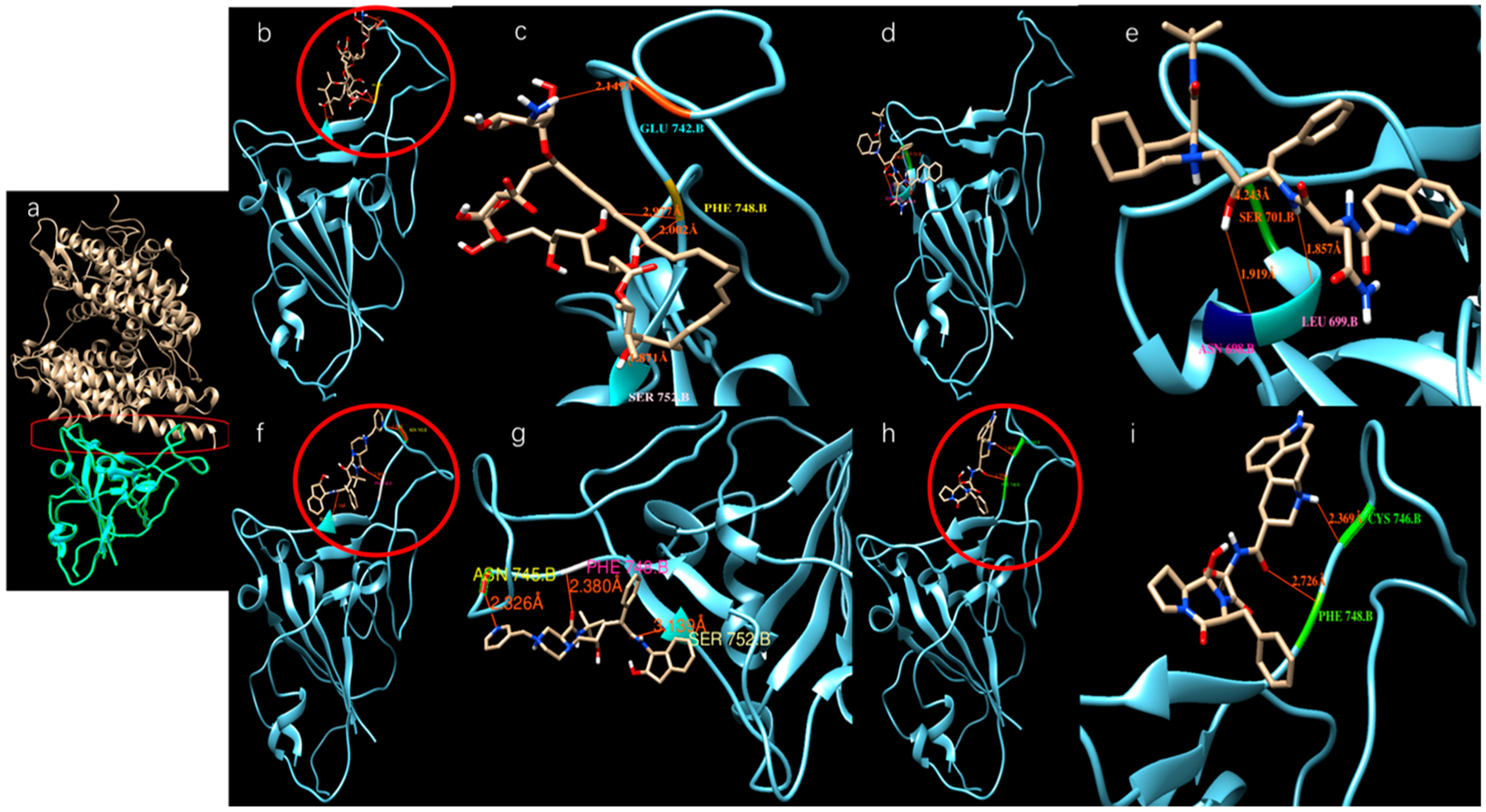
Potential active site selection and ligand-receptor interaction. (**a**) The docking site (inside the red frame) was chosen between the ACE-2 (light brown color) and SARS-CoV-2 S-protein (Cyan color); (**b**), (**c**) Amphotericin b docks onto the SARS-CoV-2 S-protein; (**d**), (**e**) Saquinavir docks onto the SARS-CoV-2 S-protein; (**f**), (**g**) Indinavir docks onto the SARS-CoV-2 S-protein; (**h**), (**i**) Ergotamine binds onto the SARS-CoV-2 S-protein.

We modeled ligands that may bind at a large docking area on the top of the S-protein that interacts with ACE-2 (red cycle in Figure 3a). Table 2 shows the binding affinities of several ligands with the highest docking scores toward the top docking side of the S-protein. Figure 3b–i shows the dockings of several ligands with the SARS-CoV-2 S-protein.

To understand whether these ligands are reasonably good inhibitors that block the interaction of the SARS-CoV-2 S-protein with ACE-2, the docking energy of the S-protein/ligand complex with ACE-2 was calculated and the results are listed in Table 3. For comparison, the docking energy between the SARS-CoV S-protein and ACE-2 was also calculated and the score was −92.7 kcal/mol, which was close to the −78.6 kcal/mol reported by Xu et al.’s work [4]. The docking energy between the SARS-CoV-2 S-protein and ACE-2 was calculated to be −82.2 kcal/mol, suggesting a slightly weaker interaction than that of the SARS-CoV S-protein with ACE-2. The observation is similar to that reported by Xu et al.’s work [4]. Table 3 shows that more than half of those ligands docking onto the SARS-CoV-2 S-protein do not significantly change the interaction of the SARS-CoV-2 S-protein with ACE-2—i.e., they are not inhibitors to block the interaction of the SARS-CoV-2 S-protein with ACE-2. However, ergotamine, amphotericin B, vancomycin, zafirlukast, and lanicor showed that once they were bound to the S-protein, the interactions of these complex with ACE-2 were no longer energetically favored interactions—i.e., these ligands acted as desired inhibitors that can efficiently block the interaction of the SARS-CoV S-protein with ACE-2. Among these, ergotamine and amphotericin b are most promising, since they demonstrate the highest docking energy to the SARS-CoV-2 S-protein (Table 2). Thus, they are strongly suggested as the core drugs for clinical trials to treat COVID-19 patients. Considering the severe and potentially lethal side effects of amphotericin b [28], ergotamine and vancomycin seem be the top choices.

## 4. Discussion

**Figure 4.**
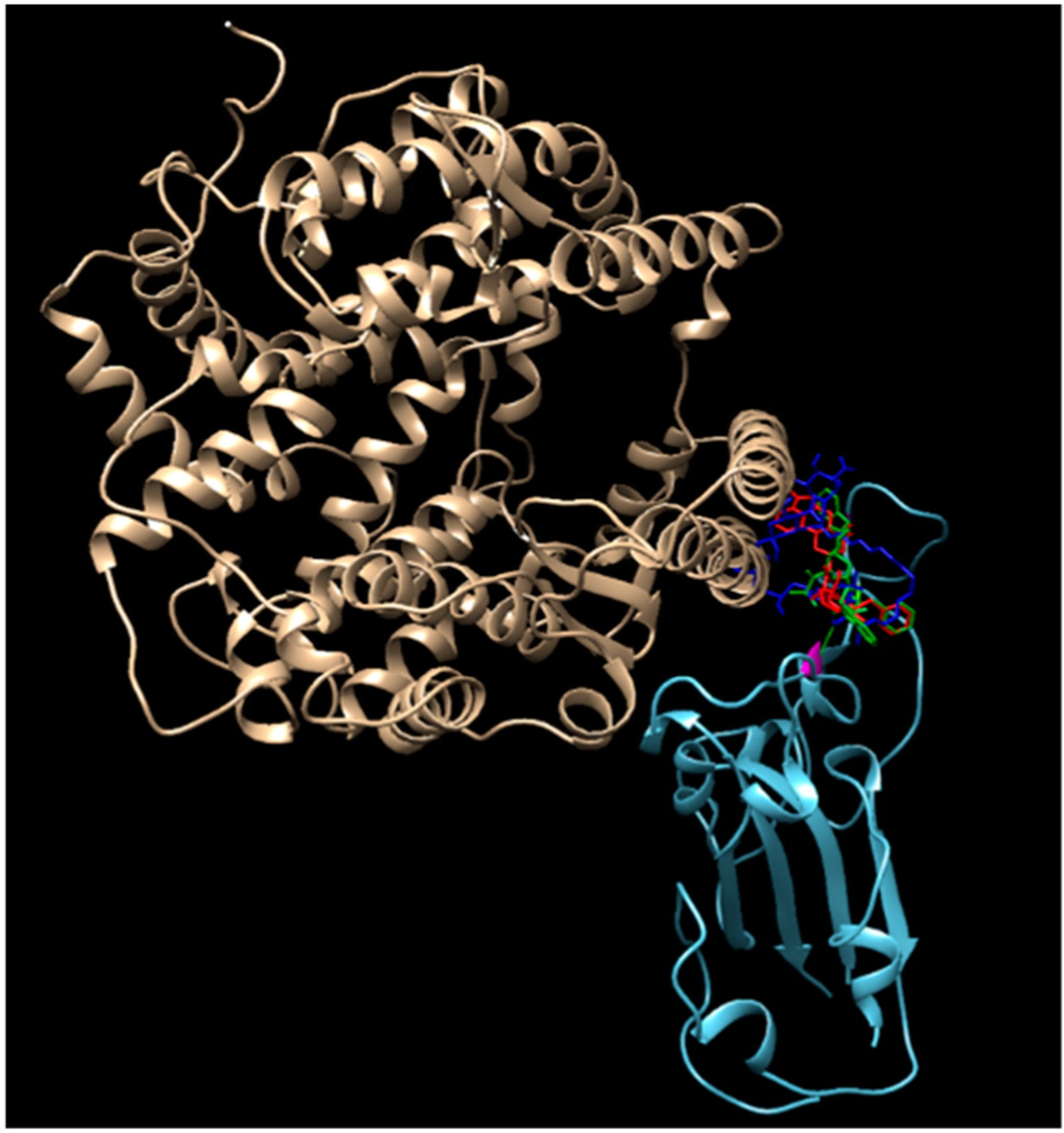
An overlay of the modeled structures of amphotericin, ergotamin, indinavir, and ACE-2 with the SARS-CoV-2 spike protein.

Disulfiram, lopinavir, and ritonavir are the three approved and active protease inhibitors against SARS and MERS. Indeed, lopinavir and ritonavir were successfully used to treat a patient in Thailand in Jan. 2020. Our results show that among these ligands, saquinavir, tadalafil, rivaroxaban, sildenafil, dasatinib, vardenafil, montelukast are most promising due to their higher docking scores (< −8.5 kcal/mol, which coresponds to < 1 μM IC50) than others. All of these scores appear better than that of the antiviral drug Lopinavir (−8.2 kcal/mol). As a comparison, the docking scores reported for lopinavir with the viral RNA polymerase is −8.3 kcal/mol [18]. It is a remarkable observation that some SARS-CoV-2 inhibitors such as indinavir could not block the interaction of the SARS-CoV-2 S-protein with ACE-2, while other inhibitors, such as ergotamine and amphotericin B, can effectively inhibit such interaction. This is somewhat confusing, since all of these three compounds dock on the same docking site that is marked by the red circles in Figure 3—the grove between an extended insertion that contains the β5/β6 strands and the receptor-binding motif (RBM) loop [28]. To comprehend what caused the significant difference, we overlaid the structures of the three docked compounds and ACE-2 on the SARS-CoV-2 spike protein in Figure 4. The comparison clearly shows that ergotamine (red) and amphotericin B (blue) extend further out toward the ACE-2 and thus effectively block the interaction of the SARS-CoV-2 spike protein with ACE-2 while indinavir (green) clings to the SARS-CoV-2 spike protein, leaving room for ACE-2 to interact with the spike protein.

## 5. Conclusion

For the inhibition of the SARS-CoV-2 3^CL^ protease, saquinavir, tadalafil, rivaroxaban, sildenafil, dasatinib, vardenafil, and montelukast are most promising due to their high docking scores (<− 8.5 kcal/mol), which were more negative than those of other ligands.

Among these that showed an excellent inhibiting property to block the interaction of SARS-CoV-2 S-protein with ACE-2 in Table 3, ergotamine, amphotericin b, and vancomycin are the most promising since they are also among the highest to bind to the SARS-CoV-2 S-protein, as shown in Table 2.

For more active results, a combination of 3^CL^ protease inhibitors and ergotamine may be considered.

## Figures and Tables

**Table 1.** Different docking scores (binding affinities) of the tested drugs for SARS-CoV-2 proteinase.

Drug Name	Docking Score (kcal/mol)	Usage
Saquinavir	−9.5	Antiretroviral drug to treat or prevent HIV/AIDS [20]
Tadalafil	−9.3	A medication used to treat erectile dysfunction (ED), benign prostatic hyperplasia (BPH), and pulmonary arterial hypertension [21]
Rivaroxaban	−9.2	An anticoagulant medication used to treat and prevent blood clots [22]
Sildenafil	−8.9	A medication used to treat erectile dysfunction and pulmonary arterial hypertension [23]
Dasatinib	−8.8	A targeted therapy used to treat certain cases of chronic myelogenous leukemia (CML) and acute lymphoblastic leukemia (ALL) [24]
Vardenafil	−8.7	A PDE5 inhibitor used to treat erectile dysfunction [25]
Montelukast	−8.5	To treat seasonal and year-round allergies [26]
Indinavir	−8.3	A component antiretroviral therapy to treat HIV/AIDS [27]
Lopinavir	−8.2	Protease inhibitor
Cortisone	−8.2	Can be used for a variety of conditions
celecoxib	−8.1	An anti-inflammation drug
Atazanavir	−8.1	An antiretroviral drug used for HIV treatment
Iressa	−7.9	A drug for cancer treatment
Darunavir	−7.7	An antiretroviral drug used for HIV treatment
Sorafenib	−7.5	A drug for cancer treatment

**Table 2.** Different docking scores of ligands for the SARS-CoV-2 S-protein.

Drug Name	Docking Score (kcal/mol)	Usage
Ergotamine	−8.8	For treatment of acute migraine attacks [29]
Amphotericin b	−8.3	An antifungal medication used for serious fungal infections and leishmaniasis [30]
Indinavir	−8.1	A component antiretroviral therapy to treat HIV/AIDS
Vancomycin	−7.7	For treatment bacterial infections [31]
Lonpinavir	−7.7	An antiretroviral, often used against HIV infections
Zafirlukast	−7.6	For the chronic treatment of asthma
Lanicor	−7.5	Used to treat heart conditions [32]
PubChem ID: 54098557	−7.5	--
Digitaline Nativelle	−7.5	For treatment of congestive heart failure, also used as angiotensin-converting enzyme (ACE) inhibitor
Rivaroxaban	−7.5	To treat and prevent blood clots [33]
Tadalafil	−7.5	To treat erectile dysfunction
Nelfinavir	−7.3	The treatment of HIV
Montelukast	−7.2	Treatment of asthma
Saquinavir	−7.1	The treatment of HIV
Carfilzomib	−7.1	Anti-cancer drug as proteasome inhibitor
Lapatinib	−7.0	Anti-cancer drug
Atovaquone	−7.0	To treat pneumocystis pneumonia, toxoplasmosis, malaria and babesia
Celecoxib	−7.0	An anti-inflammation drug
Vardenafil	−6.9	For treatment of erectile dysfunction
Dasatinib	−6.8	To treat certain cases of chronic myelogenous leukemia
Cortisone	−6.6	Can be used for a variety of conditions

**Table 3.** Docking energy of the SARS-CoV S-protein with and without ligands to human ACE-2.

Interaction of S-Protein and S-Protein/Drug Complex with ACE-2	Docking Energy (kcal/mol)
SARS-CoV S-protein (for comparison)	−92.7
SARS-CoV-2 S-protein	−82.2
SARS-CoV-2 S-protein/Ergotamine	56.4
SARS-CoV-2 S-protein/Amphotericin b	78.6
SARS-CoV-2 S-protein/Indinavir	−61.9
SARS-CoV-2 S-protein/Vancomycin	81.7
SARS-CoV-2 S-protein/Zafirlukast	52.6
SARS-CoV-2 S-protein/Lanicor	4.2
SARS-CoV-2 S-protein/Nelfinavir	−81.5
SARS-CoV-2 S-protein/Montelukast	−71.3
SARS-CoV-2 S-protein/Saquinavir	−48.2
SARS-CoV-2 S-protein/Carfilzomib	−88.1
SARS-CoV-2 S-protein/Lapatinib	−83.1
SARS-CoV-2 S-protein/Atovaquone	−68.2
SARS-CoV-2 S-protein/Celecoxib	−74.2
SARS-CoV-2 S-protein/Dasatinib	−42.3
